# Lidocaine-Loaded Hyaluronic Acid Adhesive Microneedle Patch for Oral Mucosal Topical Anesthesia

**DOI:** 10.3390/pharmaceutics14040686

**Published:** 2022-03-22

**Authors:** Tingting Zhu, Xixi Yu, Xin Yi, Xiaoli Guo, Longhao Li, Yuanping Hao, Wanchun Wang

**Affiliations:** 1 School of Stomatology of Qingdao University, Qingdao 266003, China; zhutingting_91@sina.com (T.Z.); 2020026442@qdu.edu.cn (X.G.); 2 Qingdao Stomatological Hospital Affiliated to Qingdao University, Qingdao 266001, China; kqyuxixi@yeah.net; 3 School of Electromechanical Engineering, Guangdong University of Technology, Guangzhou 510006, China; yixin@mail2.gdut.edu.cn; 4 Department of Stomatology, School of Stomatology of Weifang Medical University, Weifang 261053, China; lilonghao1013@outlook.com

**Keywords:** adhesive microneedle, lidocaine, hyaluronic acid, topical anesthesia, oral mucosal, transdermal drug delivery

## Abstract

The pain and fear caused by direct local injection of anesthetic or the poor experience with surface anesthetic cream increase the difficulty of clinical treatment for oral diseases. To address this problem, a hyaluronic acid microneedle patch (Li-HAMNs) that consists of fast-dissolving lidocaine hydrochloride (LDC)-loaded tips and a wet-adhesive backing layer made of polyvinyl alcohol (PVA)/carboxymethylcellulose sodium (CMC-Na) was fabricated to explore its potential use in dental topical anesthesia. Li-HAMNs could puncture the stratum corneum with an insertion depth of about 279 μm in the isolated porcine oral mucosal. The fast-dissolving tips could release LDC to improve the patients’ convenience and compliance. Importantly, the backing layer, which has good adhesion ability and water-absorbing properties, could surmount the contraction and extension of oral masticatory muscles and the saliva scour. In the tail flick test, the topical anesthesia efficacy of the Li-HAMNs group was much better than clinical lidocaine cream (EMLA cream, LDC, 1.2 mg) in spite of a relatively lower LDC dose with Li-HAMNs (LDC, 0.5 mg). It is believed that the proposed adhesive microneedle patch could enhance transmucosal delivery of anesthetics and thus open a new chapter in the painless treatment of oral diseases.

## 1. Introduction

Oral diseases are often accompanied by pain such as recurrent aphthous ulcer, pulpitis, apical periodontitis, trigeminal nerve pain, etc. [1]. At the same time, there will be pain during the treatment of these diseases. The use of local anesthesia has become the main means of modern oral treatment for pain and anxiety [2,3], however, the short-term pain caused by the injection of local anesthesia or induction of trypanophobia affects the effect of the treatment [4]. At the same time, fear and anxiety can aggravate chronic pain [5]. In order to reduce the pain or fear during treatment or injection of anesthesia needles, topical anesthetics such as creams, gels, and sprays are often used clinically. Topical anesthetic creams prepared with lidocaine have been widely used in various skin practices [6,7]. This is because lidocaine, with the feature of low risk of anaphylaxis, fast onset of time, and moderate duration of action, is one of the most common and effective local anesthetics in oral clinic [8]. Although topical anesthetics have the superiority of simple operation, no pain, no injury, reducing the patient’s fear of pain and avoiding injection pain [9], these topical anesthetics have difficulty in penetrating the oral mucosal barrier, therefore, surface anesthetics are sluggish to take effect [10]. In addition, there are some shortcomings such as low adhesion [11], easy to be diluted by saliva, or easy to be swallowed by child patients. Therefore, a fast-acting anesthesia product that can penetrate the physical barrier of the mucosa while being adhesive and absorbable is urgently needed in clinic.

Microneedles (MNs), which could penetrate the physical barrier of cuticle in a minimally invasive way, are a new transdermal drug delivery system [12]. MNs are divided into four categories: solid, hollow, drug loaded MNs, and dissolving MNs [13]. Among them, dissolving microneedles (DMNs) are prepared by mixing biodegradable polymers with biocompatible bioactive materials [14,15,16], and have sufficient mechanical strength to puncture the cuticle and dissolve in body fluids to release bioactive materials [17,18,19]. To date, there have been several researches on dermal anesthetic DMNs [2,4,10,19,20] and lidocaine DMNs have been used to enhance the delivery of local anesthetics to the skin [19]. Nevertheless, lidocaine DMNs have not been developed for transmucosal deliver topical anesthesia for the oral mucosa. It is well-known that oral mucosa is a highly diverse and dynamic environment. How to deliver anesthesia accurately and efficiently to the lesions and maintain their effects is, however, still a challenging problem [21].

Carboxymethylcellulose sodium (CMC-Na), as a highly water-soluble polysaccharide, has been widely used due to its biocompatibility, biodegradable good combination, and stability [22,23,24]. Polyvinyl alcohol (PVA) is widely used in the biomedical field because PVA is a water-soluble, non-toxic, and biodegradable synthetic polymer that can be mixed with polysaccharides to enhance its adhesion [25]. More importantly, PVA and CMC-Na can form stronger intermolecular hydrogen bonds. Blended films prepared by mixing PVA and CMC-Na have been studied regarding the water sorption and solubility [26,27]. The results show that the blended films had better water absorption and resistance due to the synergistic effects of molecular chain relaxation and intermolecular hydrogen bond formation between PVA and CMC-Na [28].

Considering the above factors, in this paper, we developed the desired oral mucosal topical anesthesia microneedle adhesive patch (Li-HAMNs) with the features of penetration ability, better rapid and effective painless, minimal invasion, easy operation, and wet adhesion by employing a Lego-brick-stacking-inspired fabrication strategy. Such Li-HAMNs consisted of two components: the fast-dissolving LDC-loaded tips and PVA/CMC-Na wet-adhesive backing layer, which could overcome the contraction and extension of oral masticatory muscles and the flushing effect of saliva. HA, as the main carbohydrate component of dermal extracellular matrix, which has been approved by the U.S. Food and Drug Administration (FDA) to fill soft tissue damage in 2003 [29], was chosen as the tip materials. Next, the characteristics of the fabricated Li-HAMNs including physical properties, quantitative analysis of LDC content, oral mucosa insertion experiment, in vivo drug retention, cytotoxicity test of Li-HAMNs, tail flick test, and stability evaluation were investigated. The results showed that the fabricated Li-HAMNs could achieve rapid submucosal dissolution to enhance the effect of topical anesthesia, providing a new idea for the painless treatment of oral diseases.

## 2. Materials and Methods

### 2.1. Preparation of PVA/CMC-Na Blend Film

PVA (0.4 g), CMC-Na (0.2 g), and glycerin (0.2 g) (Sinopharm Chemical Reagent Co, Ltd., Shanghai, China) were dissolved in distilled water (DW, 10 mL) and stirred at 80 °C for 15 min to obtain a uniform backing layer solution. The hot solution was poured on a 30 × 24 × 0.2 cm^3^ glass plate, and it was quickly leveled off with a rule. The glass plate was dried at room temperature for 24 h to obtain the PVA/CMC-Na blend film and was reserved at room temperature.

### 2.2. Preparation of Li-HAMNs

**Scheme 1:**
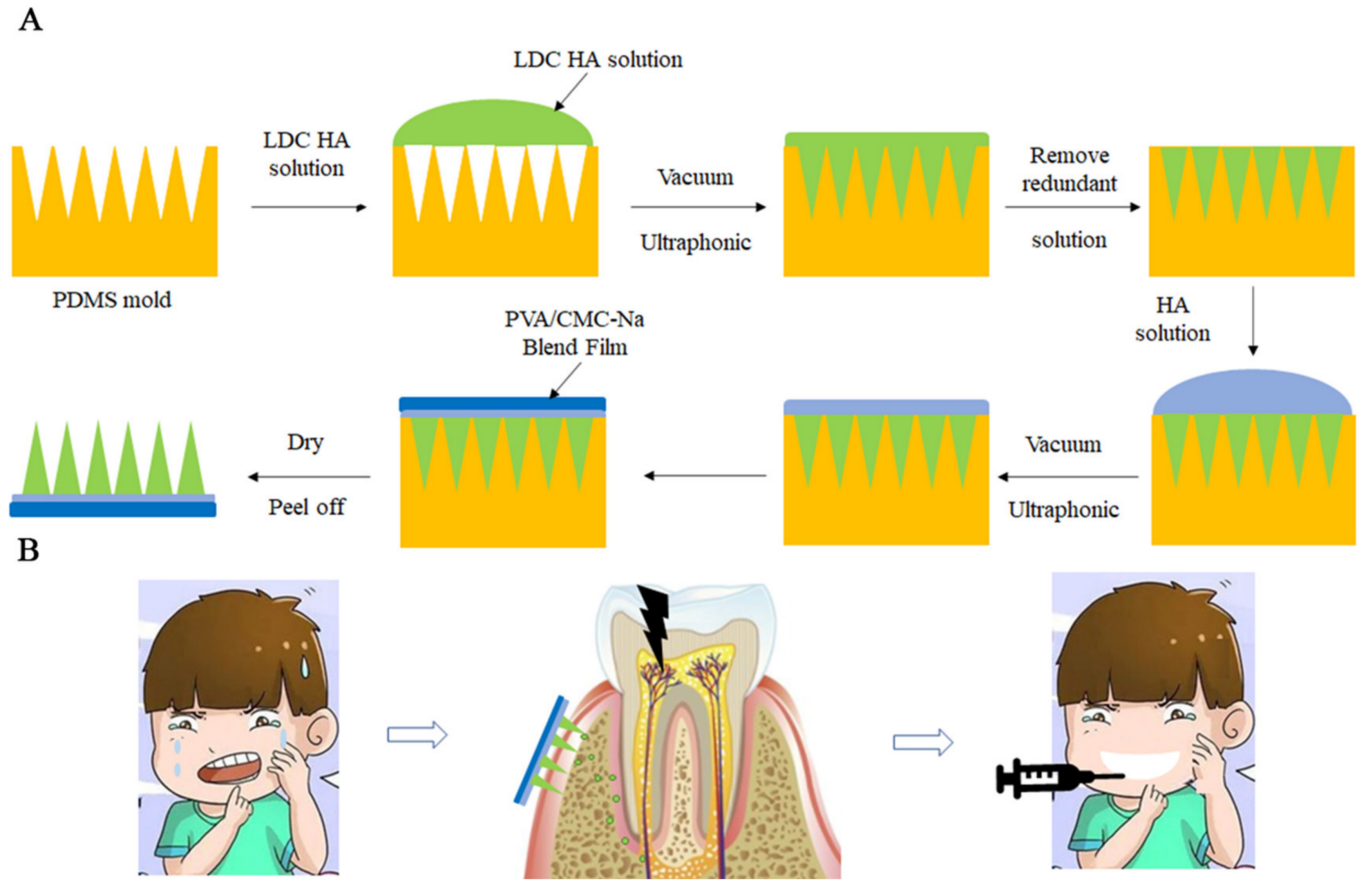
(**A**) Schematic representation of the preparation of Li-HAMNs. (**B**) The use of Li-HAMNs relieves the pain and trypanophobia caused by the injection of local anesthesia.

The polydimethylsiloxane mold (PDMS, Shenzhen Thunder Cloud Information Technology Corp, Shenzhen, China) consisted of 100 (10 × 10) microneedles with a tip-to-tip space of 700 μm was employed to fabricated the microneedles. Each needle was 280 μm in width and 700 μm in height. A total of 216.9 mg of LDC (Solarbio, Beijing, China) and 120 mg of HA (Mw = 40–100 kDa, Macklin, Shanghai, China) were dissolved in DW (4 mL) to obtain a drug-loaded solution. First, PDMS mold was injected with DW placed under vacuum for 15 min. Next, 20 mg of the drug-loaded solution was poured into each PDMS mold and placed in an ultrasonic instrument (Ningbo Xinzhi Biotechnology Co., Ltd., Ningbo, China) for 30 min to fill the cavities of the PDMS mold and form the tips of microneedles. Then, excess solution left on the surface of the PDMS mold was removed. After that, a pure HA solution (3 wt%) was added to the surface of the mold, and then the PVA/CMC-Na blend film was placed on the HA solution, and excess HA solution was removed to form the base of the microneedles. Finally, these PDMS molds were dried at room temperature for 24 h, and Li-HAMN patches were obtained by gently detaching them from the molds and kept in the desiccator containing silica gel. The preparation and application process of Li-HAMNs are shown in Scheme 1. The rhodamine B-loaded Li-HAMNs were fabricated through a similar procedure by incorporating rhodamine B to the LDC solution.

### 2.3. Characterization of Li-HAMNs

#### 2.3.1. The Thickness of the PVA/CMC-Na Blend Film

The thickness of the PVA/CMC-Na blend film was tested with a thickness gauge (PHYNIX, Neuss, Germany) and different parts of the samples were tested in parallel for 10 times. The average thickness was recorded.

#### 2.3.2. The Morphology of Li-HAMNs

The macro-morphology of Li-HAMNs was examined using a digital camera (Nikon, Tokyo, Japan). The distribution of LDC in the microneedle patch was observed by a confocal laser scanning microscope (Leica Microsystems, Wetzlar, Germany). To assess the microstructure of the blend film and Li-HAMN patches, all samples were measured using a scanning electron microscope (SEM, VEGA3, TESCAN Co., Ltd., Shanghai, China) at an acceleration voltage of 10 kV. Samples were sputter-coated with gold to increase the conductivity before imaging.

#### 2.3.3. Fourier Transform Infrared Spectroscopy (FTIR)

A Nicolet iN10 FTIR spectrometer (Thermo Fisher Scientific, Waltham, MA, USA) was utilized to characterize the FTIR spectra of the samples (i.e., PVA, CMC-Na, glycerol, PVA/CMC-Na blend film, HA, LDC, Li-HA, and Li-HAMNs) over the range of 500−4000 cm^−1^ at a scanning resolution of 2 cm^−1^ for 32 scans.

#### 2.3.4. Swelling Rate and Wettability of PVA/CMC-Na Blend Film

The PVA/CMC-Na blended film was cut into a square of 10 × 10 mm, and its initial dry mass was recorded as m_1_. Then, at specific time intervals, the excess water on the surface of the blend film was removed with filter paper, and its mass was recorded as m_s_. There were three parallel samples in each group. The swelling rate can be calculated according to the following formula:

(1) $$\mathrm{swelling}\;\mathrm{rate}=(m_{s}\;-\;m_{1})/m_{1}\;\times \;100\%$$

At room temperature, the static contact angle of the blend film was measured using a contact angle instrument (Biolin Scientific Co., Ltd., Gothenburg, Sweden). The volume of the droplet was 4 μL, and each sample was measured at least three times.

#### 2.3.5. Adhesive Performance of the PVA/CMC-Na Blend Film In Vitro

Fresh buccal mucosa of five-month-old landrace pigs was obtained from a local slaughterhouse. In the Li-HAMN adhesion strength test, the buccal mucosa and muscle layer were uniformly and smoothly retained.

In order to detect the influence of the saliva scouring on the blend film, we carried out the flush test [30]. Briefly, the blended film was cut into squares of 10 × 10 mm. The pig oral mucosa was hydrated in artificial saliva (5 mL) for 5 min. The pig oral mucosa was wiped with a sterile cotton ball and the blended film sample was placed on the oral mucosa. The blended film was moistened with artificial saliva (0.5 mL). The mucosal tissues were rinsed by constant running water (110 mL/min).

#### 2.3.6. Mechanical Strength of Li-HAMNs

A universal testing machine (WDW-5G, Shandong, China) was used to assess the mechanical property of the Li-HAMN patch as described in the previous literature [31]. The PVA/CMC-Na blended film was cut into strips (20 × 10 mm), and the stretching rate was 10 mm/min. Li-HAMNs were placed on the metal platform of the universal tensile machine, and the pressure probe was pressed down at a constant speed of 0.1 mm/s.

### 2.4. Drug Loading of Microneedle Patch

A piece of Li-HAMN was dissolved in DW (20 mL) for 3 min. The maximum absorption wavelength of LDC was found at 254 nm. LDC content in Li-HAMNs were characterized by UV–Vis spectroscopy (Shanghai Metash Instruments Co., Ltd., Shanghai, China) using a standard curve method. LDC concentration is calculated according to the standard curve.

### 2.5. Cytotoxicity Test of Li-HAMNs

To examine the biocompatibility of Li-HAMNs, the cell counting kit 8 (CCK-8, Absin Bioscience Inc., Shanghai, China) assay was conducted following our previous method [32,33]. LDC powder, PVA/CMC-Na blended film, and Li-HAMNs were dissolved in complete Dulbecco’s modified Eagle medium (DMEM) containing 10% fetal bovine serum (FBS) and 1% penicillin/streptomycin (Biological Industries, Israel) double antibiotics, respectively. The obtained material stock solution was diluted into five groups (25, 50, 100, 200, and 300 μg/mL). Human oral keratinocyte (HOK) cells were cultured using complete DMEM (containing 10% FBS and 1% penicillin/streptomycin double antibiotics) in a humidified incubator (WIGGENS Co., Ltd., Germany) at 37 °C with 5% CO_2_. After HOK cells were seeded in 96-well plates with a density of 4.0 × 10^3^ cells/well and cultured for 24 h, DMEM medium was replaced by the liquid extract (100 μL) of each group. The cells treated with fresh DMEM medium were set as the control group. After incubation for 2, 12, 24, and 48 h, the culture medium was replaced by 100 μL fresh DMEM containing 10% of CCK-8. The plate was incubated for 2 h in a humidified incubator. Finally, the OD values of the medium were tested at 450 nm using a microplate reader (SynergyH1/H1M, Bio-Tek Co., Ltd., Beijing, China), and cell viability could be calculated as the previous literature [31].

### 2.6. Animal

The animal protocol was examined by the ethics committee of Qingdao Stomatological Hospital affiliated with Qingdao University (contract grant 2021KQYX018). The research content and design of the project were in line with the ethical norms. All experiments followed the instructions of the Laboratory Animal Care and Use Guide. Male adult Sprague Dawley rats (~150 g) and New Zealand white rabbits (~2 kg) were purchased from the Jinan Pengyue Laboratory Animal Breeding Center (Jinan, China) and kept with free access to food and water.

### 2.7. Mucosa Insertion Test and LDC Retention In Vivo

In vitro insertion experiment of Li-HAMNs was conducted using the porcine oral mucosa. Li-HAMNs were vertically and uniformly pressed on porcine oral mucosa for 3 min, then removed. The tissue was fixed in the formalin solution, and observed by hematoxylin and eosin (H&E) staining.

In order to evaluate the effect of Li-HAMNs’ transmucosal administration, Li-HAMNs were applied to rabbit tongue abdomen mucosa. Rabbits were anesthetized with an injection of sodium pentobarbital (50.0 mg/kg). Li-HAMNs were pressed into rabbit tongue abdomen mucosa for 3 min and then removed. The photograph was taken with a macro camera. After euthanizing the rabbits, tongue tissue was taken and homogenized. The obtained specimens were quantitatively analyzed by high performance liquid chromatography (HPLC, LC-10A; Shimadzu Corp., Kyoto, Japan, *n* = 3). EMLA cream (STADA, Bad Vilbel, Germany) was used as the positive group. 

The safety of the local application of the Li-HAMNs’ patch was evaluated by testing the oral mucosa of mouse irritation following treatment. Li-HAMNs were applied to the oral mucosa of mouse for 3 min and then removed. Photographs were taken at 0 (immediate), 5, 10, and 30 min after the removal of Li-HAMNs to determine mucosal healing. After 24 h, the treated sites were dissected, fixed, and stained by a standard H&E staining procedure. The slides were scanned and analyzed by microscopic evaluation.

### 2.8. Tail-Flick Test

The water bath method was used to detect the tail-flick test as described in the reported literature [34]. Briefly, the detection time points were recorded immediately after Li-HAMNs and EMLA cream used for 3 min (0, 2, 5, 6, and 7 min, *n* = 6). Among them, the applicational dosage of EMLA cream was 60 mg, in which the content of LDC was 1.2 mg and the content of LDC in a patch of Li-HAMNs was 0.5mg. The rat was placed on the operating table with a mousetrap. After the rat adapted, its tail was immersed in a 45 °C water bath, and the time was recorded. When the rat’s tail twisted violently, or the tail was thrown out of the water, the timer was stopped and the time was recorded. Data were expressed as percentage of maximum effect (%MPE) and the areas under the curves were calculated.

### 2.9. Stability Evaluation

For Li-HAMNs stored at room temperature for six months, the stability of the Li-HAMNs patch was evaluated comprehensively by observing the morphology, assessing the ability of mucosal insertion, and testing the stability of LDC. The morphological changes were observed by macro camera photography. The mucosal insertion ability of Li-HAMNs was tested using isolated porcine oral mucosa, and the insertion depth was observed by H&E staining. The storage stability of LDCs was tested by UV–Vis spectroscopy.

### 2.10. Statistical Analysis

All values were expressed as the mean ± standard deviation (SD) and were compared by two-tailed *t*-tests for a comparison of two groups and one-way ANOVA for several groups after the normality test. The statistical analysis was performed with GraphPad Prism 8.0.

## 3. Results and Discussion

### 3.1. Preparation and Characterization of Li-HAMNs

According to the different requirements of topical anesthesia, the anesthesia radiation range is different. Therefore, the area, size, and length of Li-HAMNs should be carefully adjusted for different topical anesthesia requirements. The preparation and application process of Li-HAMNs are shown in Scheme 1. The Li-HAMNs consist of fast-dissolving tips made of HA and LDC, and a backing layer made of PVA and CMC-Na.

#### 3.1.1. Morphology and Drug Loading of Li-HAMNs

**Figure 1:**
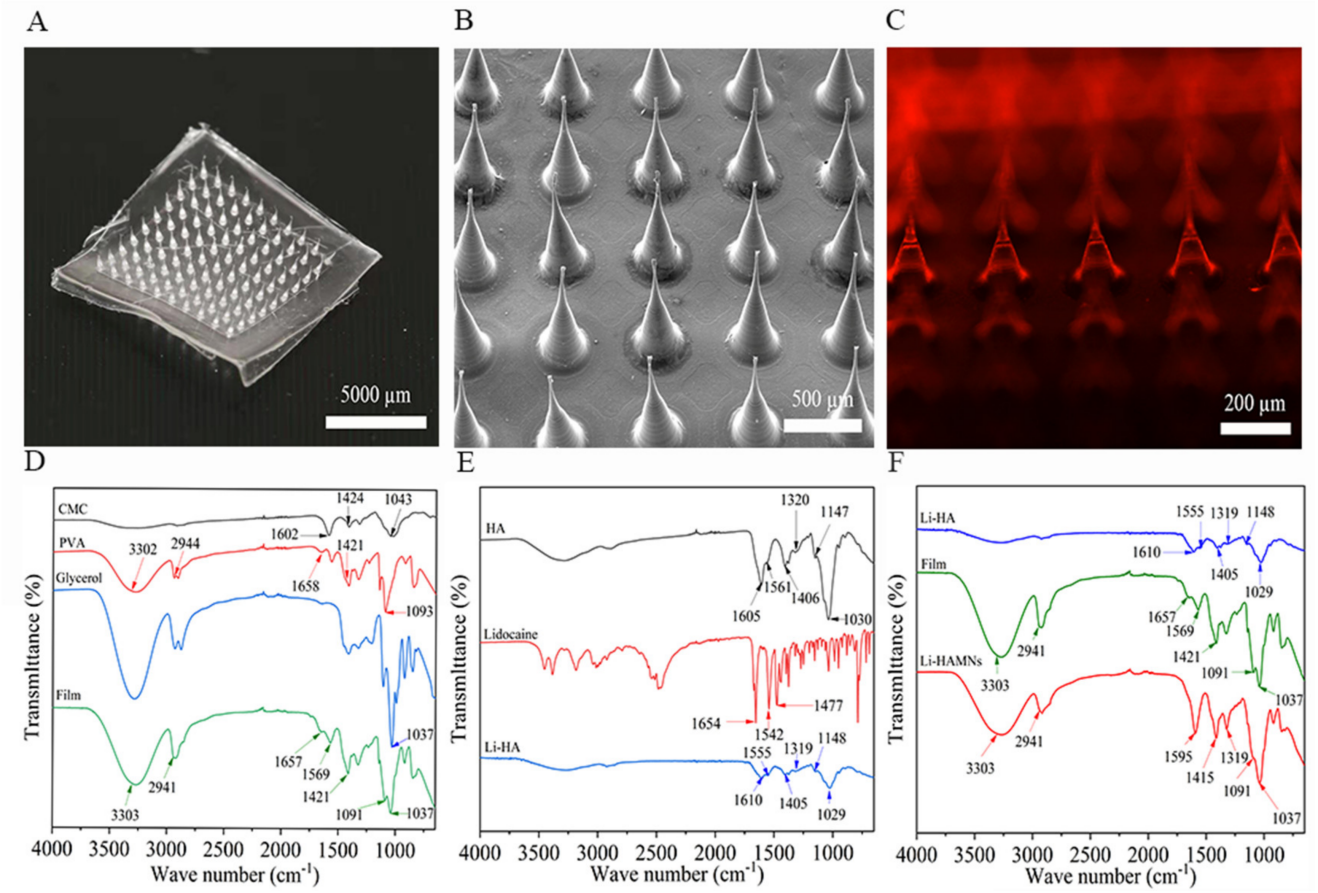
Evaluation of the physical properties of the blended film and Li-HAMNs. (**A**) The photograph of the Li-HAMN patch. Scale bar, 5000 µm. (**B**) SEM images of the Li-HAMN array. Scale bar, 500 µm. (**C**) Confocal micrographs of fluorescent Li-HAMNs. Scale bar, 200 µm. (**D**) FTIR spectrum of CMC-Na, PVA, glycerol, and blended film. (**E**) FTIR spectrum of HA, lidocaine, and Li-HA. (**F**) FTIR spectrum of Li-HA, blended film, and Li-HAMNs.

As shown in Figure 1A, LDC-loaded dissolving HA microneedles consisted of 100 (10 × 10) pyramidal needles. The fabricated microneedles had the identical structure of a conical shape. Based on the change in oral mucosa thickness (about 70 μm in the gingiva, and over 500 μm in the buccal) [35,36] and the gradual increase of pain with MN length from 700 μm to 1000 μm [37], we selected a needle with a height of 700 μm. SEM of the Li-HAMNs showed no obvious bending and breaking (Figure 1B). However, the height of the Li-HAMNs obtained in this study was 686 ± 2 μm. The decrease in dimensions may be attributed to the water evaporation during the drying process, leading to solidification contraction of the HA [29]. Importantly, the presence of saliva can affect the dissolution of microneedles. A backing layer is required to insulate the infiltration of saliva. As shown in the SEM image (Supplementary Materials, Figure S1), the surface of the blended film made of PVA and CMC-Na was dense and had no holes, which was conducive to prevent the infiltration of saliva. The tearing edge of the blended film was continuous and had a distinct interface. Furthermore, Rhodamine B was chosen as the model drug to visualize the distribution of the drug in the microneedles. Laser scanning confocal fluorescence microscopy revealed that the red fluorescence of Rhodamine B was evenly distributed at the tips of the microneedle (Figure 1C). LDC content in HA microneedles was detected by UV–Vis spectroscopy and the loading amount of LDC was 494 ± 5 µg per microneedle patch. Additionally, previous studies have shown that the 0.5 mm denture base had low foreign body sensation [38]. The thickness of the PVA/CMC-Na blended film was 93 ± 14 µm and the foreign body sensation was negligible in the oral cavity.

#### 3.1.2. FTIR Analysis of the Li-HAMNs

Since the fabrication process of microneedles was designed with simple dissolution, injection, and drying, the prepared Li-HAMNs should contain all the materials used. This was confirmed with the FTIR spectra of PVA, CMC-Na, glycerol, blended film, HA, LDC, Li-HA, and Li-HAMNs, as shown in Figure 1. The FTIR spectrum of the blended film is shown as Figure 1D. The FTIR spectra for CMC-Na illustrates band at 1602 cm^−1^ refer to the C=O bond of the carboxylate ionic groups while asymmetric bending vibration of the methyl group from CH_3_O appeared at 1424 cm^−1^. The stretching vibration of the C–O–C group of the pyranose ring had a broad peak centered at 1043 cm^−1^ [39]. The spectrum of PVA showed a peak at 3302 cm^−1^, which was attributed to O–H group stretching. The C–H from alkyl groups was observed at 2944 cm^−1^. The peak at 1658 cm^−1^ (from acetate group remaining in PVA) was attributed to C=O in PVA, while PVA displayed a peak at 1421 cm^−1^, which could be attributed to CH_2_ bending [40,41]. The PVA showed a band at 1093 cm^−1^, representing C–OH stretching [42]. The glycerol spectrum had absorption bands at 1037 cm^−1^ [43]. The characteristic peaks of the blended film include the characteristic peaks of CMC-Na, PVA, and glycerin. However, the C=O bond of carboxylate ionic groups at 1602 cm^−1^ of CMC-Na was red-shifted to 1569 cm^−1^, indicating that the C=O bond in the blended film was coordinated. Nevertheless, the FTIR spectrum of the blended film (Figure 1D) was prepared without sacrificing the biologically active functional groups in the ligand structure during the cross-linking reaction.

The FTIR spectrum of the Li-HA made of LDC and HA is shown as Figure 1E. HA displayed absorption bands at 1406 cm^−1^ and 1605 cm^−1^ that were attributed to carboxylate asymmetric stretching vibration and carboxylate symmetric stretching, respectively [44]. The HA skeleton showed peaks at 1147 and 1030 cm^−1^, which were C−O−C stretching vibration [45]. The amide bands of HA found peaks at 1561 and 1320 cm^−1^ [44]. The LDC spectrum had a sharp band at 1654 cm^−1^, which was the carbonyl group (C=O) stretching of the amide group [16,46,47,48]. Bands at 1477 and 1542 cm^−1^ were C–N stretching. The bond with higher inductive effect (O–C–N) caused the characteristic band with higher capacity at 1542 cm^−1^ [16,46,47,48]. The characteristic peaks of Li-HA included all the characteristic peaks of HA, but not the characteristic peaks of LDC. This result may be due to the uniform mixing of the drug and excipients [49,50].

According to Figure 1F, in the FTIR spectrum of Li-HAMNs, it could be found that the C=O bond of carboxylate ionic groups at 1610 and 1555 cm^−1^ for Li-HA and at 1657 and 1569 cm^−1^ for the blended film disappeared. A strong peak related to the C=O bond of carboxylate ionic groups appeared at 1595 cm^−1^. Therefore, it could be determined that this indicated the presence of more active groups and adsorption sites about the C=O bond in Li-HAMNs. The bands at 1148 and 1029 cm^−1^ disappeared, indicating that the combination of materials has removed C–O–C. The infrared spectrum curve of Li-HAMNs was closer to the infrared spectrum of the blended film, which may be due to the fact that the transmittance of the blended film was worse than that of Li-HA, which had a more significant impact on the transmittance of Li-HAMNs.

#### 3.1.3. Swelling Performance and Wettability Test of PVA/CMC-Na Blended Film

**Figure 2:**
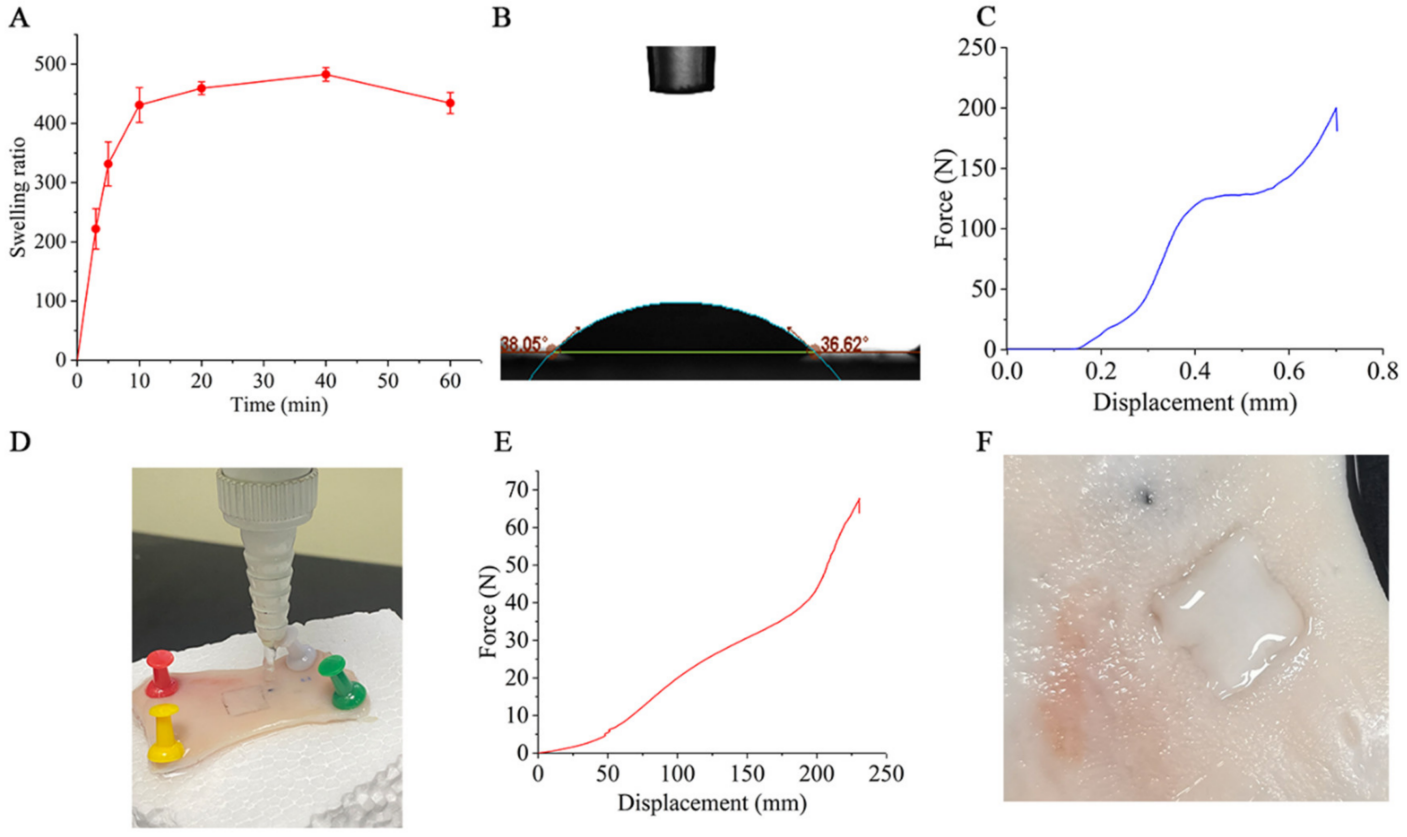
Evaluation of the physical properties of the PVA/CMC-Na blended film and the force curve of Li-HAMNs. (**A**) Results of the swelling performance of the blended film. (**B**) The static contact angle of the blended film surface. (**C**) The force curve of Li-HAMNs analyzed by the universal testing machine. (**D**) The photo of the flush test of the blended film. (**E**) The tensile stress curve of blend film analyzed by the universal testing machine. (**F**) Shape changes of artificial salivary infiltration of Li-HAMNs.

The swelling property is mainly used to characterize the water absorption capacity of membrane materials. The weight change of the blended film in artificial saliva (PH = 6.8) at different times is shown in Figure 2A. The swelling property of the blended film increased rapidly in the first 10 min, which indicates that the blended film had strong absorption for local saliva.

Furthermore, the wettability of the blended film was tested, and the results are shown in Figure 2B. The static contact angle of the blended film was 37.63 ± 0.81°, indicating that the blended film was hydrophilic. This result is consistent with our expectation that the backing layer can block and absorb saliva, matching the swelling properties of the membrane.

#### 3.1.4. Mechanics Strength of Li-HAMNs

As shown in Figure 2C, during the compression process, the curve of the entire Li-HAMNs patch (10 × 10) showed a sharp increase in force at about 0.146 mm of deformation. Subsequently, the stress of the microneedle entered the plateau stage, and finally, the force dropped suddenly and the breaking force was about 200 N. This study required that Li-HAMNs should have sufficient mechanical strength to penetrate the mucosa without mechanical failure. Studies have confirmed that the force required for microneedles to penetrate the skin is 12 N [37], whereas for mucosa, 10 N is sufficient as the cuticle of the oral mucosa is thinner than the skin [51]. The height and diameter of the Li-HAMNs chosen for this study were based on [37]. Although the number of Li-HAMNs differed from the reference, the literature specifies that a force of 10 N can penetrate the mucosa, a process that requires all needle tips to touch the mucosa. This means that a force of 10 N at the tip of the needle can penetrate the mucosa without fracture, whereas the Li-HAMNs prepared in this study could be subjected to a force of 200 N before fracture occurs. This indicates that our Li-HAMNs can penetrate the mucosa. At the same time, the results of mucosal insertion experiments, in which images and H&E staining revealed a single needle hole, confirmed the adequate strength of the Li-HAMNs.

The strength of the oral muscle group is mainly contraction and extension. This requires sufficient adhesive strength between the microneedle patch and the oral mucosal to prevent shedding. Therefore, the adhesion strength of the blended film was conducted according to previous literature. As shown in Figure 2D, the blended film did not peel from the pig oral mucosa until the flow rate reached 120 mL/min within 3 min, while for most adults, the flow rate of saliva was around 0.5 mL/min [51]. In addition, the tensile strength of the blended film was presented as a J-shaped curve with an ultimate tension value around 63 N (Figure 2E). These results indicated that the Li-HAMNs patch was strong enough to withstand the internal dynamics of oral tissue and saliva washing. In order to further confirm the adhesive performance, Li-HAMNs were pressed into the pig oral mucosa and wetted with artificial saliva to observe their shape changes (Figure 2F). When the backing layer of the Li-HAMNs were sufficiently wetted, the blended film formed a closed environment to isolate saliva from the microneedle tips. These results confirmed that the prepared Li-HAMNs had sufficient mechanical strength and good adhesion as the oral mucosal surface anesthesia patch.

### 3.2. Cytotoxicity Test of Li-HAMNs

**Figure 3:**
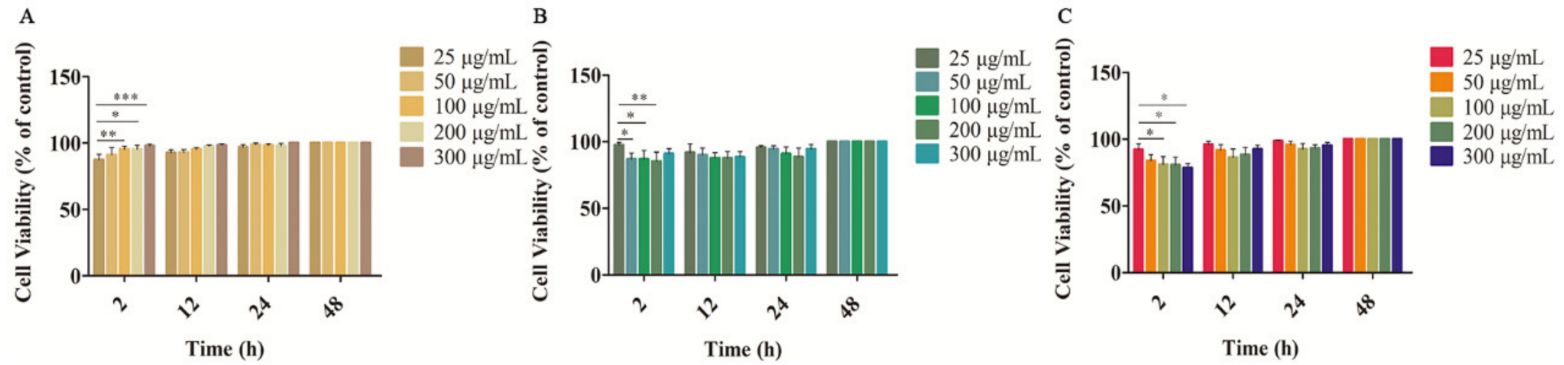
Cell viability of HOK cells after treatment with the PVA/CMC-Na blended film (**A**), lidocaine powder (**B**), and Li-HAMNs (**C**) (* *p <* 0.05, ** *p <* 0.01, *** *p <* 0.001).

In our study, the safety and biocompatibility of the blended film, lidocaine powder, and Li-HAMNs were evaluated by the CCK-8 assay using HOK cells. These tests were conducted using a material-conditioned medium for cell culture. As shown in Figure 3A, there were statistically significant differences between 25 µg/mL and other groups after incubation for 2 h. However, the cell viability of all groups exhibited more than 90%, indicating the blended film prepared in this study had good biocompatibility. In addition, with the extension of incubation time, the proliferation of HOK cells was promoted, which may be due to the nutrition provided by the polysaccharides of CMC-Na [24].

For pure lidocaine powder and Li-HAMNs, there were statistically significant differences between 25 µg/mL and other groups (Figure 3B,C). The inhibitory effect of LDC occurred after the medium was replaced for 2 h, which might be due to the biological half-life of LDC in humans [52]. The materials used to prepare Li-HAMNs have a wide range of applications in both the biomedical and medical fields, especially HA and PVA, which are approved by the U.S. FDA. PVA and CMC-Na are backing layers and non-invasive materials. On the whole, the cell viability of all groups was higher than 85%, confirming the excellent biocompatibility of the microneedles. Preclinical trials are needed to further verify the toxicity of Li-HAMNs patches before clinical application.

### 3.3. Mucosa Insertion Test and LDC Retention In Vivo

**Figure 4:**
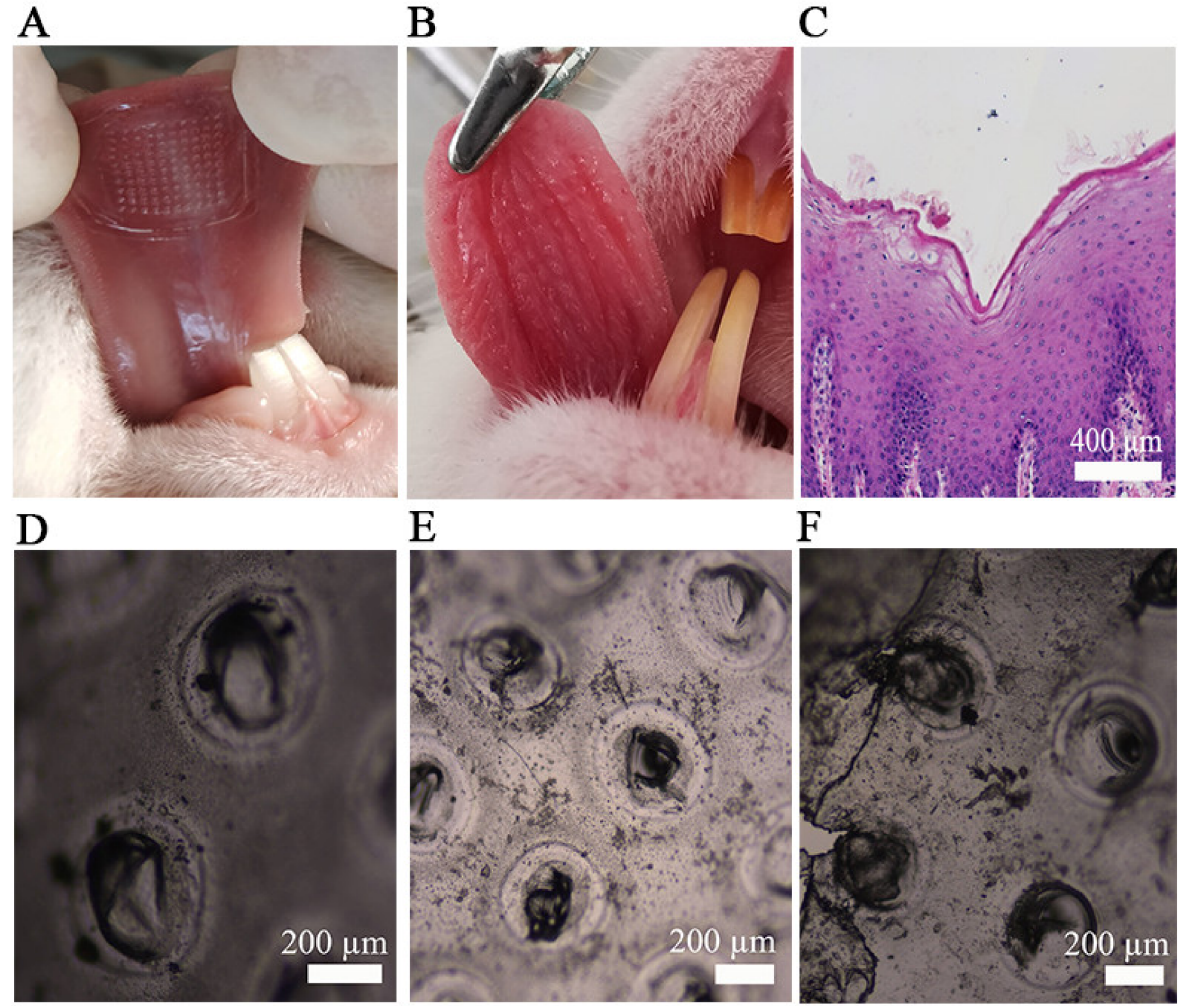
The results of the mucosa insertion test. (**A**) Image of the rabbit tongue abdomen mucosa treated with Li-HAMNs. (**B**) The photograph of the rabbit tongue abdomen mucosa surface after insertion of Li-HAMNs. (**C**) Image of H&E staining section of pig oral mucosa after the application of Li-HAMNs. (**D**–**F**) Image of Li-HAMNs after rabbit tongue abdomen mucosa dissolution under an optical microscopy image for 1 min (**D**), 2 min (**E**), and 3 min (**F**).

The Li-HAMNs were applied to porcine oral mucosa and tongue tissue of rabbit to further confirm the insertion capability. Porcine oral mucosa is often used as a biological barrier due to its similar composition, histology, and permeability to human oral mucosa [53]. In order to facilitate observation, Li-HAMNs without blended films was used in this experiment. As shown in Figure 4A, the rabbit tongue abdomen mucosa showed an array of MNs (10 × 10) corresponding to the Li-HAMNs insertion sites, confirming that the obtained Li-HAMNs were strong enough to break the oral mucosa barrier. Figure 4B shows a photograph of the rabbit tongue abdomen mucosa after the removal of Li-HAMNs, and the pinhole of Li-HAMNs could be observed with naked eyes. In order to further verify the depth of the MNs penetrated into the mucosa, H&E staining was performed using the porcine oral mucosa perforation by microneedles (Figure 4C). The deep cavities were clearly observed in pig oral mucosa, which further confirmed that the Li-HAMNs could pierce through the stratum corneum and then be embedded into the oral mucosa. The results of this study were consistent with those of Byeong-min Lee et al. [10]. Figure 4D–F shows the microscopic images of Li-HAMNs removed after 1, 2, and 3 min in vivo, respectively. The Li-HAMNs melted gradually within 3 min.

Furthermore, the tongue tissue of rabbit was used to determine LDC retention in the tongue tissue. EMLA cream was used as the positive group. The results showed that LDC content in tongue tissues in the Li-HAMNs group was twice that of the EMLA cream group, which indicated that the Li-HAMNs could deliver a greater amount of LDC into the submucosa, therefore achieving rapid and efficient administration and anesthesia of the oral mucosa.

**Figure 5:**
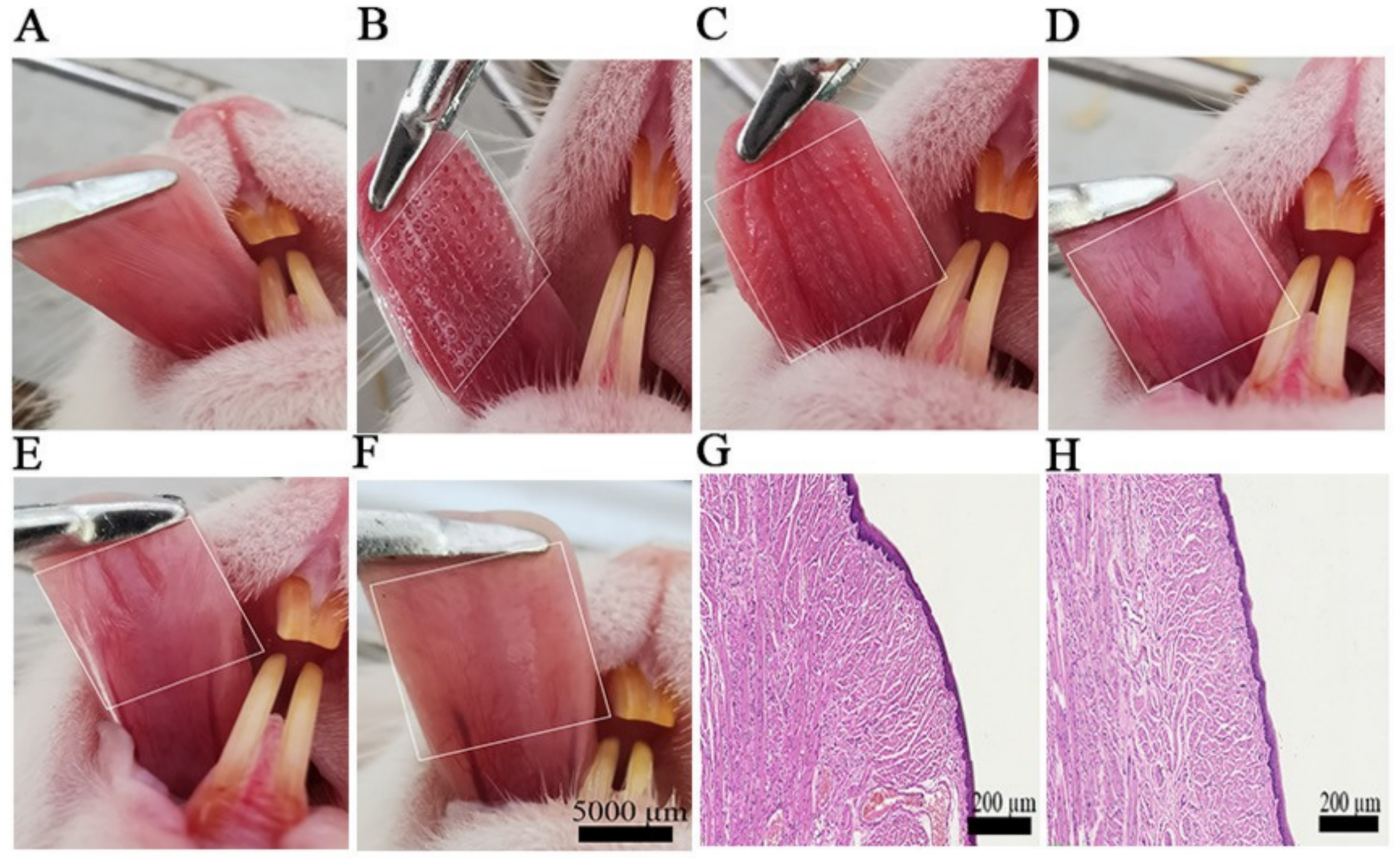
The mucosa recovery behavior after the application of Li-HAMNs. (**A**) Original mice oral cavity mucosa. (**B**) Li-HAMN treatment for 3 min. (**C**–**F**) Images at different time points after the removal of Li-HAMNs: (**C**) 0 min, (**D**) 5 min, (**E**) 10 min, and (**F**) 30 min. The white dotted bordered rectangles represent the oral cavity mucosa area treated with Li-HAMNs. (**G**,**H**) H&E staining images of mucosa untreated and treated with Li-HAMNs, respectively (*n* = 3).

Li-HAMNs were designed as a minimally invasive transmucosal drug delivery device, therefore, its damage to the mucosa was investigated. Mice without edema or erythema on the mucosa were utilized for the experiment (Figure 5A–F). After application of Li-HAMNs for 5 min, the punctured mucosa showed well-defined erythema and rapidly alleviated after 10 min (Figure 5E). After 30 min, the application site recovered its intact original state (Figure 5F). There was no visible irritation observed on the mucosa treated with Li-HAMNs compared to the untreated mucosa. 

After 24 h, the mucosa surrounding the microneedle penetration sites were dissected and prepared for histological examination. Compared to the untreated mucosa (Figure 5G), there were no overt infiltrated inflammatory cells observed on the mucosa after insertion of Li-HAMNs (Figure 5H), indicating that the applications of Li-HAMNs did not induce significant inflammation in the mucosa.

### 3.4. Tail-Flick Experiment

**Figure 6:**
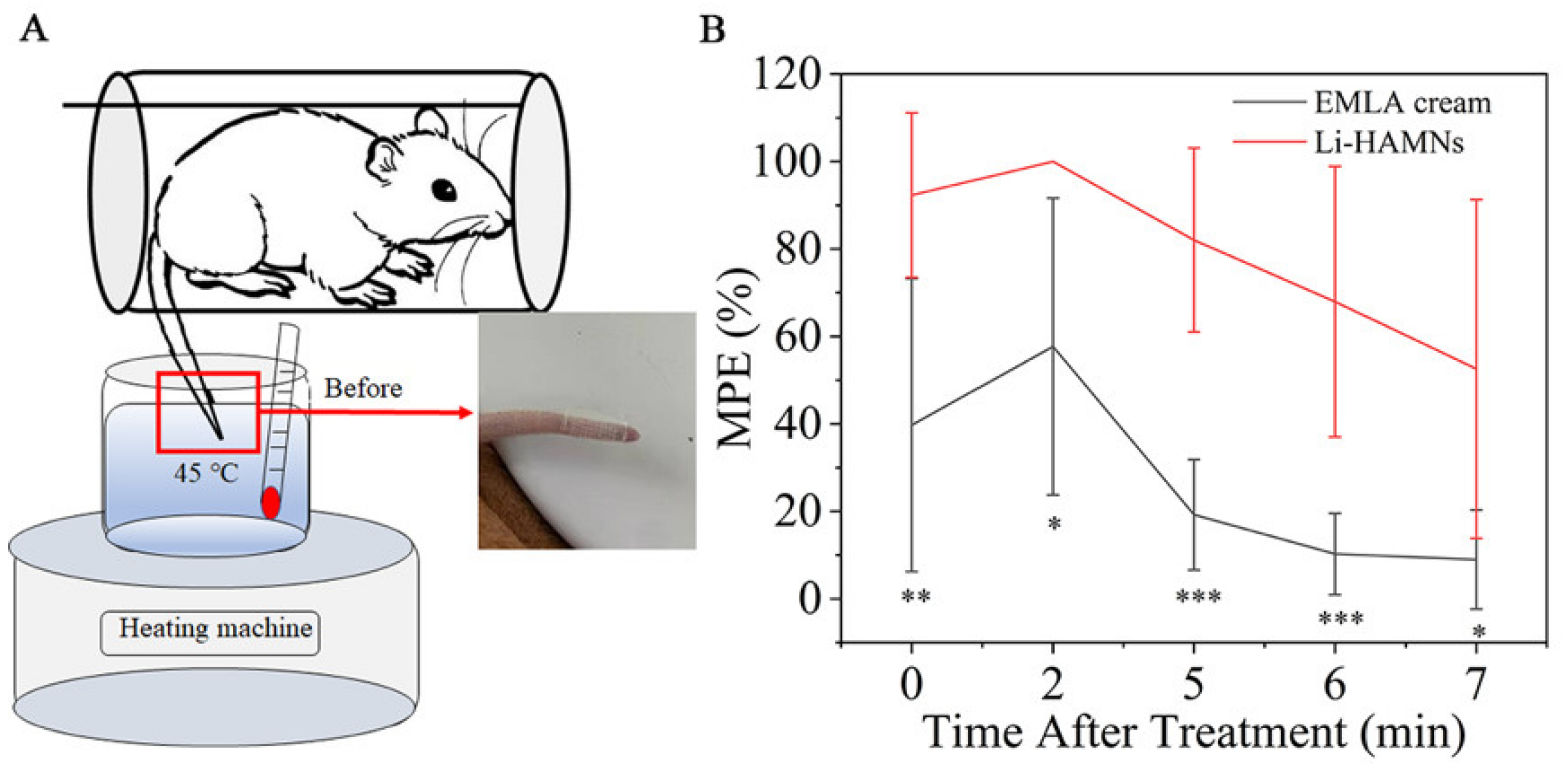
Anesthetic effect (tail-flick experiment) in rats treated with Li-HAMNs. (**A**) Schematic diagram of the water bath method of the tail-flick experiment. (**B**) Quantization of the anesthetic effect over MPE in rats treated with Li-HAMNs and EMLA cream. Data are mean ± SEM (*n* = 6). Statistical analyses were carried out by one-way ANOVA plus Tukey post hoc (* *p* < 0.05, ** *p* < 0.01, *** *p* < 0.001).

The tail-flick experiment was designed to evaluate the anesthetic effect of Li-HAMNs (Figure 6A). Li-HAMNs and EMLA cream were applied for 3 min. The test performed immediately after 3 min of EMLA cream application was in accordance with the requirements of the clinical guidelines and was comparable to the test conditions for microneedles. The results were quantified over %MPE, as shown in Figure 6B. Li-HAMNs group showed about 92% MPE, and the positive control group of EMLA cream gave 40% MPE after treatment with Li-HAMNs and EMLA cream, respectively. Both the Li-HAMNs (100% MPE) and EMLA cream (57% MPE) groups achieved the best anesthetic effect after two min, and then decreased over time. The anesthesia microneedle patch is used for topical anesthesia prior to local anesthesia, so the tail-flick experiment was performed immediately after 3 min of its effect. The anesthetic effect of Li-HAMNs is still better than that of EMLA cream, even though there are microholes in microneedle administration. These results confirmed that the topical anesthesia effect of Li-HAMNs was significantly better than the EMLA cream, which was in line with the hypothesis of our study. The results were verified again with the results of the mucosa insertion test and LDC retention in vivo, which confirmed that the surface anesthesia effect of Li-HAMNs was better than that of EMLA cream.

### 3.5. Stability Evaluation

The Li-HAMNs used for the stability evaluation were stored at room temperature for six months. There was no significant difference in morphology of Li-HAMNs (Figure S2A) after storage for six months. Li-HAMNs were inserted into the isolated porcine oral mucosa for 3 min and then removed. H&E-stained images (Figure S2B) showed that deep cavities were clearly observed in the porcine oral mucosa. These results indicated that the Li-HAMNs still had enough strength to penetrate the mucosa. Such results are particularly important for the clinical application of the products made. The content of LDC was reduced to 400 ± 38 µg per microneedle patch due to oxidized products [4]. However, the purity of LDC was higher than 80% after six months of storage.

## 4. Conclusions

Li-HAMNs are an effective local mucosal anesthetic drug delivery system with a fast onset time and the ability to achieve oral mucosal topical anesthesia. The prepared Li-HAMNs in this study meet the requirements of oral clinical application, which has the characteristics of rapid and painless administration, water resistance, and adhesion, which overcomes the disadvantages of the pain of the direct local injection of anesthetic drugs or ingestion, and an uncomfortable taste of surface anesthesia ointment. According to the results of the in vivo drug retention of Li-HAMNs, Li-HAMNs delivered two-fold lidocaine more than EMLA cream into the oral mucosa with a long residual time. In addition, the results of the cytotoxicity test of Li-HAMNs showed that Li-HAMNs met the requirements of oral topical anesthesia administration. In the tail-flick test, the topical anesthesia efficacy of the Li-HAMNs group was much better than EMLA cream (LDC, 1.2 mg) despite a relatively lower LDC dose with Li-HAMNs (LDC, 0.5 mg). Li-HAMNs can overcome the limitations of injection pain and topical anesthesia ointment in the oral clinical field and become a novel painless and adhesive local topical anesthesia method.

## Data Availability

All data available are reported in the article.

## Supplementary Materials

The following supporting information can be downloaded at: https://www.mdpi.com/article/10.3390/pharmaceutics14040686/s1, Figure S1: The force curve of blend film analyzed by the universal testing machine.; Figure S2: Test of Li-HAMNs after 6 months of storage.

## References

[1] Scrivani S.J., Spierings E.L. (2016). Classification and Differential Diagnosis of Oral and Maxillofacial Pain. Oral Maxillofac. Surg. Clin. N. Am..

[2] Seeni R.Z., Zheng M., Lio D.C., Wiraja C., Mohd Yusoff M.F., Koh W.T., Liu Y., Goh B.T., Xu C. (2021). Targeted Delivery of Anesthetic Agents to Bone Tissues using Conductive Microneedles Enhanced Iontophoresis for Painless Dental Anesthesia. Adv. Funct. Mater..

[3] Herberger K., Krause K., Maier K., Zschocke I., Radtke M., Augustin M. (2012). Local anesthetic effects of Lidocaine cream: Randomized controlled trial using a standardized prick pain. J. Dermatolog. Treat..

[4] Yang H., Kang G., Jang M., Um D.J., Shin J., Kim H., Hong J., Jung H., Ahn H., Gong S. (2020). Development of Lidocaine-Loaded Dissolving Microneedle for Rapid and Efficient Local Anesthesia. Pharmaceutics.

[5] Ochsner K.N., Ludlow D.H., Knierim K., Hanelin J., Ramachandran T., Glover G.C., Mackey S.C. (2006). Neural correlates of individual differences in pain-related fear and anxiety. Pain.

[6] Negi P., Singh B., Sharma G., Beg S., Katare O.P. (2015). Biocompatible lidocaine and prilocaine loaded-nanoemulsion system for enhanced percutaneous absorption: QbD-based optimisation, dermatokinetics and in vivo evaluation. J. Microencapsul..

[7] Sharma G., Kamboj S., Thakur K., Negi P., Raza K., Katare O.P. (2017). Delivery of Thermoresponsive-Tailored Mixed Micellar Nanogel of Lidocaine and Prilocaine with Improved Dermatokinetic Profile and Therapeutic Efficacy in Topical Anaesthesia. AAPS PharmSciTech.

[8] Babaie S., Ghanbarzadeh S., Davaran S., Kouhsoltani M., Hamishehkar H. (2015). Nanoethosomes for Dermal Delivery of Lidocaine. Adv. Pharm. Bull..

[9] Abou-Okeil A., Rehan M., El-Sawy S.M., El-Bisi M.K., Ahmed-Farid O.A., Abdel-Mohdy F.A. (2018). Lidocaine/β-cyclodextrin inclusion complex as drug delivery system. Eur. Polym. J..

[10] Lee B.M., Lee C., Lahiji S.F., Jung U.W., Chung G., Jung H. (2020). Dissolving Microneedles for Rapid and Painless Local Anesthesia. Pharmaceutics.

[11] Wang Y., Liu Y., Zhang X., Liu N., Yu X., Gao M., Wang W., Wu T. (2021). Engineering Electrospun Nanofibers for the Treatment of Oral Diseases. Front. Chem..

[12] Larrañeta E., McCrudden M.T., Courtenay A.J., Donnelly R.F. (2016). Microneedles: A New Frontier in Nanomedicine Delivery. Pharm. Res..

[13] Jin X., Zhu D.D., Chen B.Z., Ashfaq M., Guo X.D. (2018). Insulin delivery systems combined with microneedle technology. Adv. Drug Deliv. Rev..

[14] Nayak A., Das D.B. (2013). Potential of biodegradable microneedles as a transdermal delivery vehicle for lidocaine. Biotechnol. Lett..

[15] Yang H., Kim S., Kang G., Lahiji S.F., Jang M., Kim Y.M., Kim J.M., Cho S.N., Jung H. (2017). Centrifugal Lithography: Self-Shaping of Polymer Microstructures Encapsulating Biopharmaceutics by Centrifuging Polymer Drops. Adv. Healthc. Mater..

[16] Kochhar J.S., Lim W.X., Zou S., Foo W.Y., Pan J., Kang L. (2013). Microneedle integrated transdermal patch for fast onset and sustained delivery of lidocaine. Mol. Pharm..

[17] Dangol M., Yang H., Li C.G., Lahiji S.F., Kim S., Ma Y., Jung H. (2016). Innovative polymeric system (IPS) for solvent-free lipophilic drug transdermal delivery via dissolving microneedles. J. Control. Release.

[18] Lee C., Kim H., Kim S., Lahiji S.F., Ha N.Y., Yang H., Kang G., Nguyen H., Kim Y., Choi M.S. (2018). Comparative Study of Two Droplet-Based Dissolving Microneedle Fabrication Methods for Skin Vaccination. Adv. Healthc. Mater..

[19] Ito Y., Ohta J., Imada K., Akamatsu S., Tsuchida N., Inoue G., Inoue N., Takada K. (2013). Dissolving microneedles to obtain rapid local anesthetic effect of lidocaine at skin tissue. J. Drug Target..

[20] Zhang Y., Brown K., Siebenaler K., Determan A., Dohmeier D., Hansen K. (2012). Development of lidocaine-coated microneedle product for rapid, safe, and prolonged local analgesic action. Pharm. Res..

[21] Serpe L., Jain A., de Macedo C.G., Volpato M.C., Groppo F.C., Gill H.S., Franz-Montan M. (2016). Influence of salivary washout on drug delivery to the oral cavity using coated microneedles: An in vitro evaluation. Eur. J. Pharm. Sci..

[22] Elshazly E.H., Yu L., Zhang Y., Wang H., Chen K., Zhang S., Ke L., Gong R. (2019). Fabrication of folate-phytosterol-carboxymethyl cellulose nanoparticles derived from plant material as carrier of anticancer drug. Micro Nano Lett..

[23] Maver T., Kurečič M., Pivec T., Maver U., Gradišnik L., Gašparič P., Kaker B., Bratuša A., Hribernik S., Stana Kleinschek K. (2020). Needleless electrospun carboxymethyl cellulose/polyethylene oxide mats with medicinal plant extracts for advanced wound care applications. Cellulose.

[24] Allafchian A., Hosseini H., Ghoreishi S.M. (2020). Electrospinning of PVA-carboxymethyl cellulose nanofibers for flufenamic acid drug delivery. Int. J. Biol. Macromol..

[25] El Fawal G., Hong H., Song X., Wu J., Sun M., Zhang L., He C., Mo X., Wang H. (2020). Polyvinyl Alcohol/Hydroxyethylcellulose Containing Ethosomes as a Scaffold for Transdermal Drug Delivery Applications. Appl. Biochem. Biotechnol..

[26] El-Sayed S., Mahmoud K.H., Fatah A.A., Hassen A.D. (2011). DSC, TGA and dielectric properties of carboxymethyl cellulose/polyvinyl alcohol blends. Phys. B Condens. Matter.

[27] Zhang L., Zhang G., Lu J., Liang H. (2013). Preparation and Characterization of Carboxymethyl Cellulose/Polyvinyl Alcohol Blend Film as a Potential Coating Material. Polym.-Plast. Technol. Eng..

[28] Zhu J., Li Q., Che Y., Liu X., Dong C., Chen X., Wang C. (2020). Effect of Na_2_CO_3_ on the Microstructure and Macroscopic Properties and Mechanism Analysis of PVA/CMC Composite Film. Polymers.

[29] Zhu Z., Luo H., Lu W., Luan H., Wu Y., Luo J., Wang Y., Pi J., Lim C.Y., Wang H. (2014). Rapidly dissolvable microneedle patches for transdermal delivery of exenatide. Pharm. Res..

[30] Wu T., Cui C., Huang Y., Liu Y., Fan C., Han X., Yang Y., Xu Z., Liu B., Fan G. (2020). Coadministration of an Adhesive Conductive Hydrogel Patch and an Injectable Hydrogel to Treat Myocardial Infarction. ACS Appl. Mater. Interfaces.

[31] Xie Y., Wang H., Mao J., Li Y., Hussain M., Zhu J., Li Y., Zhang L., Tao J., Zhu J. (2019). Enhanced in vitro efficacy for inhibiting hypertrophic scar by bleomycin-loaded dissolving hyaluronic acid microneedles. J. Mater. Chem. B.

[32] He Y., Zhao W., Dong Z., Ji Y., Li M., Hao Y., Zhang D., Yuan C., Deng J., Zhao P. (2021). A biodegradable antibacterial alginate/carboxymethyl chitosan/Kangfuxin sponges for promoting blood coagulation and full-thickness wound healing. Int. J. Biol. Macromol..

[33] Hao Y., Zheng W., Sun Z., Zhang D., Sui K., Shen P., Li P., Zhou Q. (2021). Marine polysaccharide-based composite hydrogels containing fucoidan: Preparation, physicochemical characterization, and biocompatible evaluation. Int. J. Biol. Macromol..

[34] Ribeiro L., Franz-Montan M., Alcântara A., Breitkreitz M.C., Castro S.R., Guilherme V.A., Muniz B.V., Rodrigues da Silva G.H., de Paula E. (2020). Hybrid nanofilms as topical anesthetics for pain-free procedures in dentistry. Sci. Rep..

[35] Alves P., Alves T., Pegoraro T.A., Costa Y.M., Bonfante E.A., de Almeida A. (2018). Measurement properties of gingival biotype evaluation methods. Clin. Implant. Dent. Relat. Res..

[36] Brogden K.A., Squier C., Ebrary I. (2011). Human Oral Mucosa: Development, Structure and Function. Br. J. Surg..

[37] Di Carla Santos S., Fávaro-Moreira N.C., Abdalla H.B., Augusto G., Costa Y.M., Volpato M.C., Groppo F.C., Gill H.S., Franz-Montan M. (2021). A crossover clinical study to evaluate pain intensity from microneedle insertion in different parts of the oral cavity. Int. J. Pharm..

[38] Wada T., Takano T., Tasaka A., Ueda T., Sakurai K. (2016). Evaluation of participants’ perception and taste thresholds with a zirconia palatal plate. J. Prosthodont. Res..

[39] El-Newehy M.H., El-Naggar M.E., Alotaiby S., El-Hamshary H., Moydeen M., Al-Deyab S. (2016). Preparation of biocompatible system based on electrospun CMC/PVA nanofibers as controlled release carrier of diclofenac sodium. J. Macromol. Sci. A.

[40] Andrade G., Barbosa-Stancioli E.F., Piscitelli Mansur A.A., Vasconcelos W.L., Mansur H.S. (2006). Design of novel hybrid organic-inorganic nanostructured biomaterials for immunoassay applications. Biomed. Mater..

[41] Kumar A., Negi Y.S., Bhardwaj N.K., Choudhary V. (2012). Synthesis and characterization of methylcellulose/PVA based porous composite. Carbohyd. Polym..

[42] Jamnongkan T., Kantarot K., Niemtang K., Pansila P.P., Wattanakornsiri A. (2014). Kinetics and mechanism of adsorptive removal of copper from aqueous solution with poly(vinyl alcohol) hydrogel. Trans. Nonferr. Met. Soc. China.

[43] Wongsasulak S., Tongsin P., Intasanta N., Yoovidhya T. (2010). Effect of glycerol on solution properties governing morphology, glass transition temperature, and tensile properties of electrospun zein film. J. Appl. Polym. Sci..

[44] Gilli R., Kacuráková M., Mathlouthi M., Navarini L., Paoletti S. (1994). FTIR studies of sodium hyaluronate and its oligomers in the amorphous solid phase and in aqueous solution. Carbohydr. Res..

[45] Carneiro J., Döll-Boscardin P.M., Fiorin B.C., Nadal J.M., Farago P.V., Paula J.P. (2016). Development and characterization of hyaluronic acid-lysine nanoparticles with potential as innovative dermal filling. Braz. J. Pharm. Sci..

[46] Kevadiya B.D., Joshi G.V., Mody H.M., Bajaj H.C. (2011). Biopolymer–clay hydrogel composites as drug carrier: Host–guest intercalation and in vitro release study of lidocaine hydrochloride. Appl. Clay. Sci..

[47] Sawant P.D., Luu D., Ye R., Buchta R. (2010). Drug release from hydroethanolic gels. Effect of drug’s lipophilicity (logP), polymer-drug interactions and solvent lipophilicity. Int. J. Pharm..

[48] Abu-Huwaij R., Assaf S., Salem M., Sallam A. (2007). Mucoadhesive dosage form of lidocaine hydrochloride: I. Mucoadhesive and physicochemical characterization. Drug Dev. Ind..

[49] Patel M.R., Patel R.B., Parikh J.R., Patel B.G. (2015). Formulation consideration and skin retention study of microemulsion containing tazarotene for targeted therapy of acne. J. Pharm. Investig..

[50] Wang Y., Wang X., Wang X., Song Y., Wang X., Hao J. (2019). Design and Development of Lidocaine Microemulsions for Transdermal Delivery. AAPS PharmSciTech.

[51] Lee I.C., Lin W.M., Shu J.C., Tsai S.W., Chen C.H., Tsai M.T. (2017). Formulation of two-layer dissolving polymeric microneedle patches for insulin transdermal delivery in diabetic mice. J. Biomed. Mater. Res. A.

[52] Olkkola K.T., Isohanni M.H., Hamunen K., Neuvonen P.J. (2005). The effect of erythromycin and fluvoxamine on the pharmacokinetics of intravenous lidocaine. Anesth. Analg..

[53] Franz-Montan M., Serpe L., Martinelli C.C., da Silva C.B., Santos C.P., Novaes P.D., Volpato M.C., de Paula E., Lopez R.F., Groppo F.C. (2016). Evaluation of different pig oral mucosa sites as permeability barrier models for drug permeation studies. Eur. J. Pharm. Sci..

