# Supplementary material for: Lidocaine-Loaded Hyaluronic Acid Adhesive Microneedle Patch for Oral Mucosal Topical Anesthesia
Source: Pharmaceutics. 2022 Mar 22;14(4):686. doi: 10.3390/pharmaceutics14040686 (PMC9025765; doi:10.3390/pharmaceutics14040686)
Supplement: Supplementary file 1 [file pharmaceutics-14-00686-s001.zip › pharmaceutics-1610909-supplementary/pharmaceutics-1610909-supplementary.pdf]

pharmaceutics

Supplementary Materials: Lidocaine-Loaded Hyaluronic Acid Adhesive Microneedle Patch for Oral Mucosal Topical Anesthesia

Tingting Zhu, Xixi Yu, Xiaoli Guo, Longhao Li, Wanchun Wang, Yuanping Hao

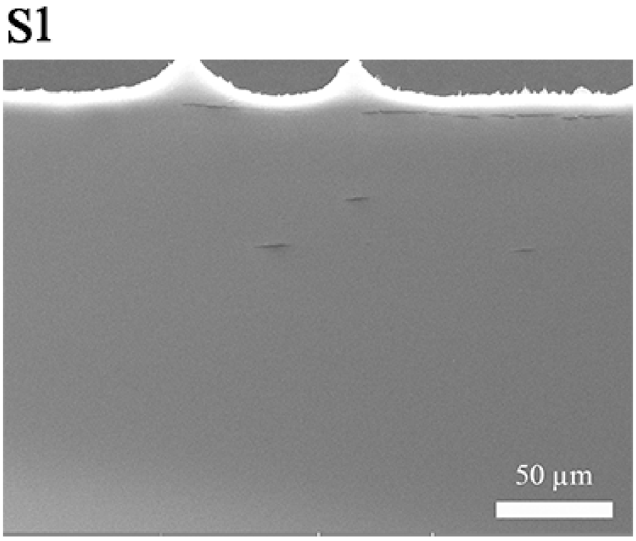

Figure S1. The force curve of blend film analyzed by the universal testing machine.

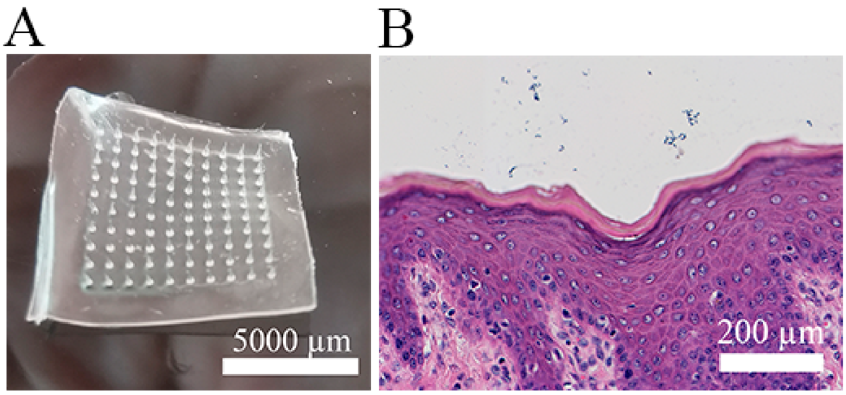

Figure S2. Test of Li-HAMNs after 6 months of storage. (A) The photograph of Li-HAMNs patch. Scale bar, 5000 µm. (B) Image of H&E staining section of pig oral mucosa after the application of Li-HAMNs.
